# Bone Marrow-Derived, Neural-Like Cells Have the Characteristics of Neurons to Protect the Peripheral Nerve in Microenvironment

**DOI:** 10.1155/2015/941625

**Published:** 2015-03-15

**Authors:** Shi-lei Guo, Zhi-ying Zhang, Yan Xu, Yun-xia Zhi, Chang-jie Han, Yu-hao Zhou, Fang Liu, Hai-yan Lin, Chuan-sen Zhang

**Affiliations:** ^1^Regenerative Medicine Center, The Second Military Medical University, Shanghai 200433, China; ^2^Nanjing Regenerative Medicine Engineering and Technology Research Center, Nanjing 210046, China; ^3^Department of Health Statistics, The Second Military Medical University, Shanghai 200433, China

## Abstract

Effective repair of peripheral nerve defects is difficult because of the slow growth of new axonal growth. We propose that “neural-like cells” may be useful for the protection of peripheral nerve destructions. Such cells should prolong the time for the disintegration of spinal nerves, reduce lesions, and improve recovery. But the mechanism of neural-like cells in the peripheral nerve is still unclear. In this study, bone marrow-derived neural-like cells were used as seed cells. The cells were injected into the distal end of severed rabbit peripheral nerves that were no longer integrated with the central nervous system. Electromyography (EMG), immunohistochemistry, and transmission electron microscopy (TEM) were employed to analyze the development of the cells in the peripheral nerve environment. The CMAP amplitude appeared during the 5th week following surgery, at which time morphological characteristics of myelinated nerve fiber formation were observed. Bone marrow-derived neural-like cells could protect the disintegration and destruction of the injured peripheral nerve.

## 1. Introduction

Peripheral nerve injury is a common clinical disease. In both the United States and Europe, tens of thousands of cases of peripheral nerve injury occur every year. Currently, the treatment of peripheral nerve defects is through the provision of a guidance channel to create a favorable microenvironment [1–3]. Although these treatments are considered successful, the results may be unsatisfactory. This is explained by the fact that the time required for reinnervation of effector muscles is much longer than the time it takes for the muscles to undergo atrophy following complete denervation. Thus, the key requirement for nerve defect repair is to slow the disintegration of peripheral nerve.

According to the current treatment of long nerve defects in peripheral nerve injuries [4, 5], we propose that “neural-like cells” may be able to protect peripheral nerve after injury. The cells are intended to be implanted into the nerve defect, such that their “neuritis” can link up with axons. This approach may prolong the time of disintegration of spinal nerves and thus reduce lesions and protect peripheral nerve.

Previously, we reported the implantation of a complex of hair follicle stem cell-derived neuron-like cells and a decellularized nerve heterograft, into a 1 cm rat sciatic nerve lesion [6]. We observed that the adult stem cell-derived neurons could survive, proliferate, and form the synaptic connections in the peripheral nerve microenvironment that promoted recovery of neurological function. However, it remains unknown whether the bone marrow-derived neural-like cells implanted into the peripheral nerve environment could be able to support the axons or others.

To determine whether the bone marrow-derived neural-like cells implanted into the peripheral nerve environment possessed neuronal characteristics, the cells were injected into the epineurium of the distal stump of transected femoral nerve. We judged the concept that cell transplantation provides nutriment that may stop peripheral nerve disintegration.

## 2. Materials and Methods

All animal care and experimental procedures were approved by the Animal Research Ethics Committee of the Second Military Medical University, Shanghai, China (permit number SYXK-2002-042).

### 2.1. Preparation of Bone Marrow-Derived Neural Tissue-Committed Stem Cells

Rabbits were anesthetized with pentobarbital sodium and 1 mL of bone marrow was harvested from the left iliac crest. The bone marrow cells were washed and cultured in D-MEM/F-12 medium (Gibco) supplemented with 10% fetal bovine serum (Gibco) and 2 mM L-glutamine (Sigma). Epidermal growth factor (EGF, Invitrogen) and basic fibroblast growth factor (bFGF, Invitrogen) were added when cells became adherent. Upon reaching 80% confluence, the cells were cultured in serum-free neurobasal medium (Gibco) supplemented with 1% N2 (Gibco) and 2% B27 (Gibco). Following the formation of cell clusters, retinoic acid (RA, 2000 nM, Sigma) and sonic hedgehog (SHH, 500 ng/mL, ProSpec-tany) were added, then the cells were used 48 hours after addition.

### 2.2. Identification of Bone Marrow-Derived Neural-Like Cells

The cells were harvested and fixed with 4% paraformaldehyde. Following permeabilization with 0.1% Triton X-100 10 min, the cells were blocked with 10% goat serum and incubated overnight at 4°C with primary antibodies against one of mouse monoclonal anti-nestin (1 : 200, Abcam), mouse IgG2*α* anti-synapsin I (1 : 200, Santa Cruz), mouse monoclonal anti-*β*-III tubulin (1 : 200, Abcam), or mouse polyclone anti-NeuN (1 : 250, Bioss). After washing, goat anti-mouse IgG FITC-conjugate secondary antibodies were incubated with the cells at room temperature for 30 minutes. Nuclei were stained with 4′, 6-diamidino-2-phenylindole (DAPI, 1 : 1000).

### 2.3. Animal Model Preparation

Rabbits were anesthetized and the saphenous nerve was separated on the lateral side of femoral artery, and the inguinal ligament was cut at its junction with the saphenous nerve. The femoral nerve was cut approximately 0.5 cm above the junction. Some neuraxial tissue was removed in the distal part of nerve, and the distal stump of the transected femoral nerve was sutured and fixed to the inguinal ligament. The cells were injected into the distal stump of the femoral nerve, following which the superficial fascia and skin were closed.

### 2.4. Animal Groups

The 20 rabbits were randomly divided into two groups (10 rabbits for each group): a control group and an experimental group. The left legs of the rabbits in the experimental group served as a sham-operated group. In the experimental group, induced neural-like cells were injected into the distal stump of the transected femoral nerve. In the control group, sterile saline was injected into the same area. In the sham-operated group, the femoral nerve was exposed but not injected.

### 2.5. Cell Transplantation

#### 2.5.1. Cell Seeding

The cells were harvested and resuspended in 10 *μ*L of serum free medium and 10 *μ*L of polyornithine to give a cell density of approximately 1 × 10^7^/mL. The cells were injected into the epineurium of the distal stump of the transected femoral nerve with a microsyringe.

#### 2.5.2. Electromyography Measurement

The compound muscle action potential (CMAP) of the rabbit quadriceps was measured at 10 time points: 3, 4, 5, 8, 11, 14, 19, 23, 24, and 26 weeks after surgery. CMAPs were recorded using a Neuropack MEB-2200 evoked potential/EMG measuring system (Nihon Koden, Japan). The intensity was 50 mA, the rate was 1 Hz, and the duration was 0.2 ms.

#### 2.5.3. Immunohistochemistry

The femoral nerves were harvested in the 26th week following surgery. The nerves were then washed and fixed. And the nerves were cut at a thickness of 7 *μ*m. The sections were dewaxed and rehydrated to enable antigen retrieval and then placed in 0.3% hydrogen peroxide in methanol for 10 minutes, blocked with 10% normal goat serum, and incubated with one of the following primary antibodies: mouse IgG2*α* anti-NF-M (1 : 150, Biolegend), mouse anti-S-100 (1 : 250, Millipore), and mouse anti-NeuN (1 : 500, Millipore). Bound antibodies were detected using the streptavidin-biotin diaminobenzidine (SABC-DAB) complex method. The nuclei of cells incubated with the NF-M and S-100 antibodies were stained with hematoxylin. The cytoplasm of the cells incubated with the NeuN antibody was stained with eosin.

#### 2.5.4. Transmission Electron Microscopy

At 26 weeks following surgery, the femoral nerves in the experimental group were harvested, prefixed, and then fixed with 1% osmium tetroxide stationary liquid. Then tissues were cut into ultrathin slices and stained with saturated uranyl acetate and lead citrate and photographed using a HITACHI H-7650 transmission electron microscope.

#### 2.5.5. Fluorescent Staining of Neuromuscular Junctions

At 26 weeks following surgery, the femoral nerve and muscle of the experimental group were harvested and sectioned as described above. Sections were blocked and treated with mouse anti-synaptophysin antibody (1 : 400, Millipore) at 4°C overnight. Bound antibody was detected using the goat anti-mouse IgG Dylight 488 (1 : 200, Jackson) secondary antibody.

### 2.6. Statistics

All data are reported as mean ± SD, and *P* value less than 0.05 was regarded as significant. The analyses were carried out using SAS (version 9.1.3). 

## 3. Results

### 3.1. Isolation, Culture, and Induction of the Bone Marrow-Derived Stem Cells

**Figure 1:**
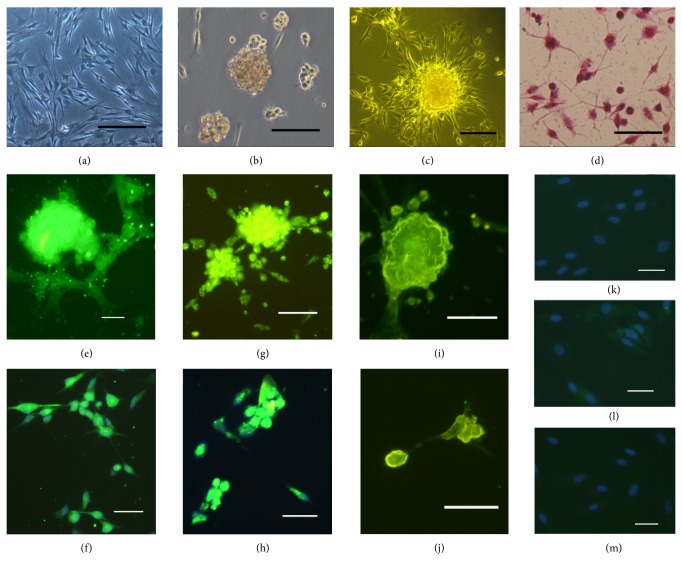
Morphology and immunofluorescence analysis of NTCSC-derived neurons. (a) Representative image of the cells at 80% confluence. (b) Representative image of cells cultured in serum-free medium. (c) The cells aggregated into groups following RA/SHH induction. (d) H&E staining revealed that the cells possessed neuron-like morphology following induction. After 48 hours of induction, the expression of Nestin ((e), (f)) and *β*-III tubulin ((g), (h)) was observed. Synapsin I was also expressed in aggregated cells (i), and NeuN was present in the nuclei (j). The uninduced cells were negative for these markers (Nestin, (k); *β*-III tubulin, (l); synapsin I, (m)). Nuclei ((f), (h), (k), (l), (m)) was staining with DAPI. Scale bar: ((c), (j), (k), (l), (m)): 100 *μ*m; ((a), (b), (d), (e), (f), (g), (h), (i)): 200 *μ*m.

Adherent bone marrow-derived stem cells began to form colonies after 5 days in culture. The cells were cultured in serum-containing medium that enabled their proliferation in vitro. After 12 days in culture, the cells had reached 80% confluence and formed irregular arrangements with a fusiform morphology. Some cells were arranged as loose spirals (Figure 1(a)). The cell density was reduced by culture in serum-free medium, in which they formed aggregates and grew in suspension (Figure 1(b)). Following RA/SHH induction, the cells became adherent and formed aggregates that extended one or more neurites (Figure 1(c)). Following gentle dispersal of the aggregated cells, H&E staining revealed that the morphology of the cells was similar to that of neurons (Figure 1(d)).

### 3.2. Immunocytochemistry

After induction with RA and SHH for 48 hours, the aggregated and adherent cells were shown by immunohistochemistry to express the neural stem cell marker, Nestin (Figures 1(e) and 1(f)), the neuron-specific marker, *β*-III tubulin (Figures 1(g) and 1(h)), and synapsin I (Figure 1(i)). The nuclei of the induced cells also expressed the neuronal nuclear antigen, NeuN (Figure 1(j)). The uninduced cells did not express nestin, *β*-III tubulin, or synapsin I (Figures 1(k), 1(l), and 1(m)).

### 3.3. Animal Models

During the early postoperative period, the motor function of the hind limbs in the control group and experimental group was obviously restricted. Later in the postoperative period, movement of the right hind limbs in the control group was abnormal and compensated by movement of the left hind limb. Hind limb movement in the experimental group was better than the control group, and movement patterns were close to normal. For the actions performed by the quadriceps, such as bounding up a flight of stairs, the experimental group was significantly better than the control group. In the experimental group, the movement of the right hind limbs was fluent and participated in locomotion.

### 3.4. Electromyography Measurement

**Figure 2:**
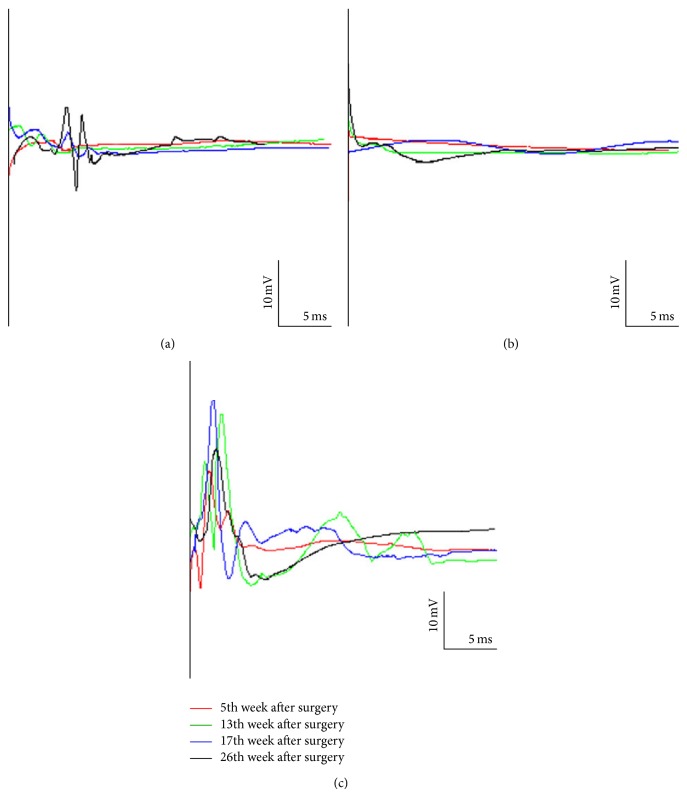
Electromyogram (EMG) of the quadriceps in the three groups. (a) Experimental group; (b) control group; (c) sham-operated group. The figure shows the compound muscle action potential (CMAP) amplitude at four time points.

The size of the recorded CMAP amplitude is positively proportional to the size and number of depolarized muscle fibers. Therefore, the degree of executive function organization can be indirectly estimated by CMAP amplitude. At all measured time points up to 3 to 4 weeks following surgery, the experimental group and control group had no detectable CMAP amplitude. However, CMAPs were weakly recorded in the experimental group in the 5th week following surgery. After that, CMAPs were continuously persistent in the experimental group. CMAPs remained undetected in the control group at all-time points. At each time point, the CMAP amplitudes in the sham-operated group were significantly greater than those in the experimental group (Figure 2). *t* test of two independent samples indicated a statistically significant difference between experimental group and control group: *t* = 2.72, *P* < 0.05 (Table 1).

### 3.5. Immunohistochemistry

**Figure 3:**
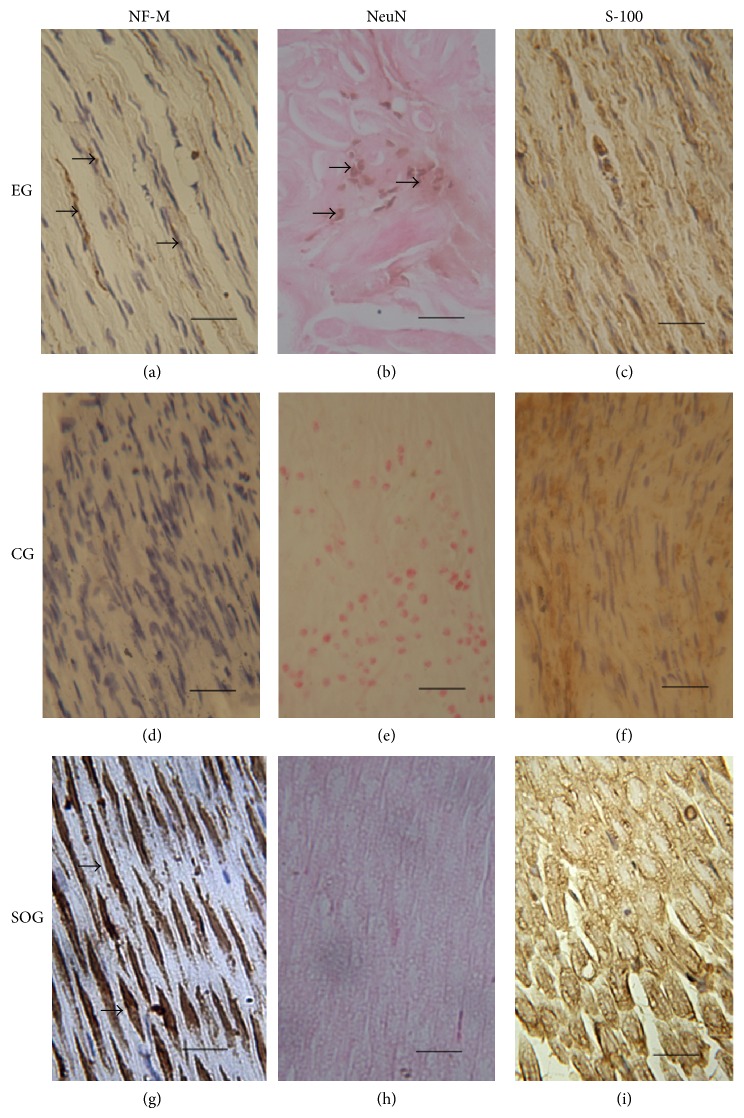
Immunohistochemistry. Positive regions (arrow) of NF-M staining were observed in the experimental group and the sham-operated group ((a), (g)), but not in the control group (d). NeuN staining was only detected in the experimental group ((b), arrow) and not the control and sham-operated groups ((h), (e)). S-100 staining in the sham-operated group was observed in regions of regular morphology (i). S100 staining in the experimental group (c) was higher than in the control group. EG: experimental group; CG: control group; SOG: sham-operated group. Scale bar: 200 *μ*m.

NF-M, NeuN, and S-100 staining of the femoral nerve sections were observed in all three groups of animals. Staining of the neuron-specific marker, NF-M, in the experimental group revealed fewer NF-M-positive cells, and thinly striped brown regions were observed between some nuclei (Figure 3(a)). NF-M-positive cells were not detected in the control group (Figure 3(d)). The largest number of NF-M-positive cells was present in the sham-operated group, in which they were regularly arranged with a ribbon-like morphology. However, the number of nuclei in the sham-operated group was lower compared to the experimental and control groups (Figure 3(g)). Staining of the glial-specific antigen, S-100, revealed many positive cells in all three groups. In the experimental group, the positive cells were irregularly shaped with a deep brown color (Figure 3(c)). In the control group, the star-shaped positive regions were scattered between nuclei (Figure 3(f)). However, in sham-operated group, the positive regions were regularly shaped and only lightly colored (Figure 3(i)). Staining of the neuron-specific nuclear antigen, NeuN, indicated that the brown positive nuclei were only detected in the experimental group (Figure 3(b)) and not in the sham-operated and control groups (Figures 3(h) and 3(e)).

### 3.6. Formation of Myelinated Nerve Fibers and Neuromuscular Junctions

**Figure 4:**
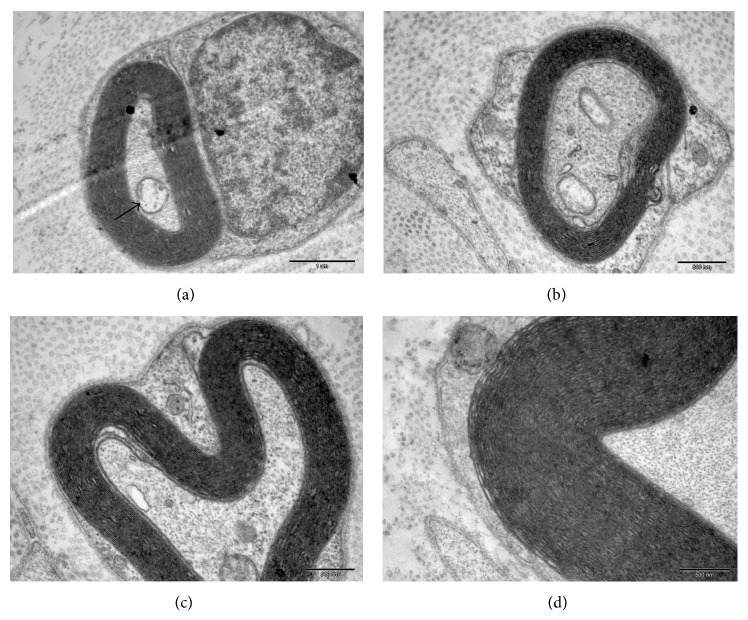
Transmission electron microscopy (TEM) of the femoral nerve from the experiment group. Axon-like structures were wrapped in myelin (a) and mitochondria were also observed (arrow). The thickness of the myelin sheath showed significant difference between the three groups ((b), (c), (d)). Scale bar: (a) 1 *μ*m; ((b)–(d)) 500 nm.

**Figure 5:**
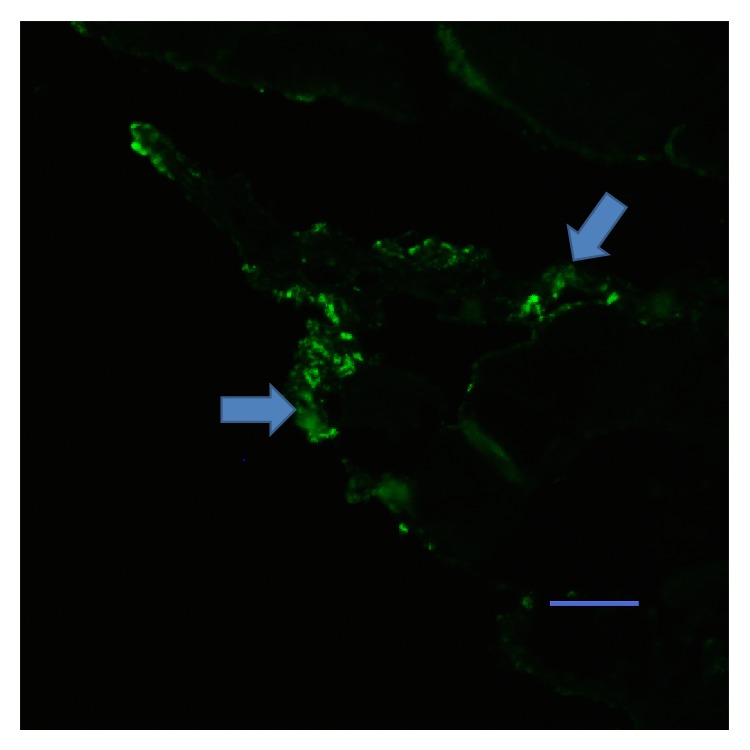
Antibody staining of neuromuscular junctions in the experimental group. The junction of nerve (upper left corner) and muscle (right hand side) was positive (arrow) for synaptophysin staining. Scale bar: 400 *μ*m.

To further verify that the immunohistochemical staining of NF-M and NeuN in the experimental group was authentic, sections of the femoral nerves from this group were examined by transmission electron microscopy at 26 weeks following surgery. In nerve cross-sections, axons or axon-like structures wrapped in an intact myelin sheath were observed. Mitochondria were also observed in these axons (Figure 4(a)). In the observed plane, the density of the myelinated nerve fibers was significantly lower than in the control group, and the thickness of myelin sheath was also significantly less (Figures 4(b), 4(c), and 4(d)). However, the junctions between nerve and muscle in the experimental group were positive for synaptophysin antibody staining (Figure 5). Synaptophysin is a synaptic vesicle glycoprotein that exists in neuromuscular junctions. These results therefore suggested that neuromuscular junctions were formed in the experimental group.

## 4. Discussion

In this study, bone marrow-derived neural-like cells were transplanted into the epineurium of the distal stump of the transected femoral nerve. Without the contribution from the new axons generated in the central nervous system, we observed changes in the quadriceps EMG in vivo, the NeuN-positive staining in the cells injection site, the presence of neuromuscular junctions by antibody staining, and increased numbers of myelinated fibers in femoral nerves. Those results showed that bone marrow-derived neural-like cells have the characterization of neuron in the injured peripheral nerve after injection, and the cells could protect the disintegration and destruction of the injured peripheral nerve.

In similar studies, embryonic stem cell-derived motor neurons, cells from the ventral spinal cord, and spinal cord neural precursor cells have been transplanted into the nerve stump. These studies demonstrated that the transplanted cells could reside in the peripheral nerve environment, make functional connections with muscle, and reduce muscle atrophy [7–11]. Our study is therefore another exciting demonstration that easily obtained and transplanted stem cells contribute to neuronal survive in the peripheral nerve environment.

Bone marrow mesenchymal stem cells (BM-MSCs) injected into the site of nerve injury can differentiate into Schwann-like cells and support nerve regeneration [12–14]. It has been postulated that BM-MSCs release nerve growth factor that stimulates Schwann cell proliferation and enhances sciatic nerve regeneration [15]. BM-MSCs may also promote the growth of axons, resulting in massive networks of branches and excess motor end plates [16, 17]. In this study, myelinated nerve fibers were still observed in the transected femoral nerve 27 weeks after surgery without stimulation of host nerve tissue. The traditional view holds that Wallerian degeneration would occur in peripheral nerves when their connection to the central nervous system is lost, such that myelinated nerve fibers disintegrate and disappear. It is known that motor neurons are not present in peripheral nerve, and the spinal ganglia are not involved in our experimental model. We therefore demonstrated that bone marrow-derived neural-like cells are capable of not only protecting axon but also protecting the Schwann cells around myelinated nerve fibers.

Other researchers have postulated that CXCR4-positive tissue-committed stem cells reside in the bone marrow, which include muscle, heart, liver, and neural tissue-committed stem cells [18, 19]. It is also believed that marrow stromal cells have the potential to differentiate into neurons [1, 20, 21]. Previous studies have shown that mesenchymal stem cells express neural progenitor cell surface markers induced by RA and fibroblast growth factor in vitro [22, 23]. Retinoic acid induced the formation of synaptic connections between MSC-derived neurons or between neurons and effectors [24, 25]. Additionally, bone marrow-derived MSCs can be induced to differentiate into dopaminergic neurons by SHH [26, 27]. In contrast, expression of the neuron-specific marker, neurofilament, may not be the result of genuine neurite extension but may rather represent cell shrinkage in response to chemical stress or cell fusion [28, 29]. Our results demonstrated that, 27 weeks after cell transplantation, complete myelin was found in the femoral nerve that had no connection with central nervous system. Because of Wallerian degeneration, myelinated nerve fibers should completely collapse. Also, motor neurons are known not to exist in peripheral nerve. We therefore propose that the injected cells have characterization of neurons and protected myelinated nerve fibers. These results demonstrate that bone marrow-derived neural-like cells could protect the disintegration and destruction of the injured peripheral nerve.

## Figures and Tables

**Table 1:** The CMAP amplitudes in three groups at each time point.

*i*	Weeks	Experimental group amp. (mV)	Control group amp. (mV)	Sham operation group amp. (mV)
1	5	1.50	0.00	16.00
2	8	1.80	0.00	25.50
3	11	0.73	0.00	12.18
4	14	1.90	0.00	12.08
5	19	3.80	0.00	25.60
6	23	5.92	0.00	15.05
7	24	7.17	0.00	20.30
8	26	11.00	0.00	20.80

Mean		4.23	0.00	18.44

Std		3.56	0.00	5.44

Data are expressed as mean ± SD. Experimental group: 4.23 ± 3.56. Control group: 0.00 ± 0.00. Sham operation group: 18.44 ± 5.44. Two independent samples of *t* test showed that there is statistical difference between experimental group and control group, *t* = 2.72, *P* < 0.05. CMAP: compound muscle action potential; SD: standard deviation.
